# The cross-cultural and chronological evolution of the kidney-bone relationship: from ancient medical insights to modern molecular endocrinology

**DOI:** 10.3389/fendo.2026.1835818

**Published:** 2026-09-24

**Authors:** Jinke Sun, Qiaoling Yuan, Peng Yu, Qiqi Wei, Yongping Qiao

**Affiliations:** Shandong Wendeng Osteopathic Hospital, Shandong, China

**Keywords:** CKD-MBD, FGF23-Klotho, gut-kidney-bone axis, kidney-bone axis, renal osteodystrophy, secondary hyperparathyroidism, traditional Chinese medicine, vitamin D metabolism

## Abstract

The kidney-bone axis constitutes a conserved bidirectional endocrine network that orchestrates skeletal remodeling and systemic mineral homeostasis. Its dysregulation underlies chronic kidney disease-mineral and bone disorder (CKD-MBD), renal osteodystrophy, secondary hyperparathyroidism, and associated vascular calcification, markedly elevating fracture and cardiovascular mortality risks. This review offers a unique cross-cultural and chronological synthesis of the kidney-bone relationship, bridging ancient medical insights with modern molecular endocrinology. Horizontally, we systematically compare the “kidney governing bones” doctrine in Traditional Chinese Medicine (TCM), ancient Greek humoral theory, and Ayurvedic Asthi Dhatu concept, revealing their shared holistic visceral-skeletal paradigm as the primitive prototype of contemporary multi-organ crosstalk, while highlighting distinct interpretive and therapeutic frameworks. Vertically, modern research is delineated into three developmental stages: (1) clinical-pathological observation of renal osteodystrophy and vitamin D-PTH disturbances (19th–early 20th century); (2) core molecular endocrine discoveries centered on the FGF23-Klotho, vitamin D, and OPG/RANKL/PTH signaling axes; and (3) expansion to novel multi-organ networks involving gut microbiota-derived uremic toxins, exosomes, and O-GlcNAc modification. We further summarize the multi-target mechanisms of kidney-tonifying TCM formulas (e.g., Zuogui Pill, Gushudan) and Western interventions, critically analyze translational bottlenecks—including biomarker limitations, stratified therapy challenges, and insufficient high-level clinical evidence—and propose integrated Chinese-Western strategies, multi-omics precision biomarkers, and cross-cultural theoretical fusion to advance personalized management of CKD-MBD and related metabolic bone diseases.

## 1 Introduction

Skeletal remodeling and systemic mineral homeostasis rely on bidirectional endocrine communication between bone tissue and the kidney, forming a conserved inter-organ regulatory axis recorded across multiple ancient medical civilizations and continuously enriched by modern clinical pathology and molecular endocrinology research (1, 2). Persistent disruption of kidney-bone crosstalk triggers CKD-MBD, senile osteoporosis and medial vascular calcification, greatly increasing long-term fracture and cardiovascular mortality (3). Existing reviews mostly focus on isolated research perspectives, lacking integrated horizontal cross-cultural comparison and vertical chronological evolution analysis, which hinders the organic integration of traditional medical empirical theories and modern targeted intervention strategies (1, 2).

### 1.1 Cross-cultural ancient medical cognition of kidney-bone correlation

The core TCM proposition “the kidney stores essence, governs bones and generates marrow” was systematically recorded in Huangdi Neijing, establishing a visceral-skeletal regulatory framework over two thousand years ago (1). The TCM kidney is not limited to anatomical renal tissue, but a composite functional system covering the HPG axis, neuroendocrine-immune network and calcium-phosphate metabolic signaling cascade (4). Sufficient kidney essence nourishes bone marrow and maintains trabecular integrity; kidney essence deficiency acts as the core intrinsic pathogenesis of osteopenia and renal osteodystrophy (1, 4, 5). Multi-omics RNA-seq and network pharmacology research verify that classic kidney-tonifying prescriptions and monomers modulate OPG/RANKL signaling to balance osteoblast and osteoclast activity, providing modern biological evidence for ancient visceral theory (5, 6).

Beyond TCM, ancient Greek humoral theory and Ayurvedic Asthi Dhatu doctrine independently constructed visceral-bone association systems, yet few systematic comparative literatures sort their homologous ideological roots and cognitive divergences (2). Most existing studies only discuss a single medical system, failing to excavate primitive endocrine prototypes hidden in cross-cultural empirical wisdom (2).

### 1.2 Three developmental stages of modern kidney-bone research

#### 1.2.1 Stage 1: clinical pathological observation stage (19th–early 20th century)

Early uremic case cohorts preliminarily defined renal osteodystrophy (ROD), verifying that progressive glomerular filtration decline independently induces skeletal lesions irrelevant to primary orthopedic diseases (7). Subsequent vitamin D research reshaped mineral metabolism cognition: renal tubular CYP27B1 is the rate-limiting enzyme generating bioactive calcitriol; nephron loss suppresses CYP27B1 activity, leading to hypocalcemia, phosphate retention and secondary hyperparathyroidism (SHPT) (8). Comprehensive pathological research on SHPT built a complete phosphate-vitamin D-parathyroid disturbance cascade (9).

#### 1.2.2 Stage 2: core molecular endocrine stage

The discovery of the FGF23-Klotho ternary complex uncovered the first bidirectional bone-kidney endocrine feedback loop (10). Osteocytes secrete FGF23 to inhibit renal phosphate reabsorption and CYP27B1 transcription, while membrane-bound renal Klotho serves as an indispensable co-receptor for FGF signal transduction (11, 12). Under CKD conditions, renal Klotho expression drops sharply, impairing tubular FGF23 signal sensitivity and triggering massive compensatory FGF23 over-secretion, forming a self-amplifying pathological vicious cycle (3, 10). Serum FGF23 rises earlier than PTH and phosphate in mild renal impairment, acting as an early diagnostic biomarker of CKD-MBD (12).

#### 1.2.3 Stage 3: multi-organ crosstalk expansion stage

Recent studies extend the kidney-bone axis to three novel regulatory networks centered on gut microbiota, exosomes and O-GlcNAc modification (13, 14). Intestinal dysbiosis causes accumulation of indoxyl sulfate and p-cresyl sulfate, activating the AhR pathway to inhibit osteogenic differentiation (7). Exosomes mediate long-distance signal exchange between kidney and bone (15); O-GlcNAc modification synchronously regulates shared injury pathways of heart, kidney and bone (14). At present, most mechanistic evidence comes from cell and rodent preclinical experiments, lacking large longitudinal human cohort validation (13).

### 1.3 Research bottlenecks & manuscript structural arrangement

Four prominent unresolved bottlenecks restrict current kidney-bone research:

Conventional serum calcium, phosphorus and intact PTH cannot distinguish high-turnover osteitis fibrosa from adynamic bone disease; the vitamin D metabolite ratio (VMR) has superior predictive value but lacks unified stage-specific clinical cutoffs (16).

SGLT2 inhibitors and anti-RANKL monoclonal antibodies exert contradictory skeletal safety effects in different renal function subgroups, with insufficient long-term RCT hard endpoint data (17, 18).

Translational research on kidney-tonifying TCM is immature, dominated by small animal experiments with scarce multi-center human clinical evidence (19).

Existing reviews lack complete chronological evolution frameworks combining cross-cultural horizontal comparison and molecular vertical combing.

Four core research objectives are proposed to fill the above gaps:

1. Horizontally compare similarities and divergences of kidney-bone doctrines among TCM, ancient Greek medicine and Ayurveda (1, 2).
2. Vertically sort three developmental phases of modern renal-skeletal research and elaborate pathological cascades of ROD, SHPT and FGF23-Klotho axis (7, 10).
3. Systematically summarize multi-target regulatory mechanisms of kidney-tonifying prescriptions, building a bridge linking TCM kidney essence theory and modern mineral endocrinology (5, 20).
4. Prospect multi-omics biomarker development and integrated Chinese-Western individualized intervention strategies (16, 21).

This review adopts six chapters: Chapter 2 Cross-cultural ancient medical theories; Chapter 3 Modern physiology and pathology of kidney-bone disorder; Chapter 4 Molecular endocrine signaling axes; Chapter 5 Clinical translation and TCM intervention; Chapter 6 Controversies and future directions. This work constructs a unified theoretical system spanning ancient empirical medicine and modern precision biomedicine.

## 2 Chapter 2 cross-cultural ancient medical theories linking kidney and bone

### 2.1 The “kidney governs bones” doctrine in traditional Chinese medicine

The TCM theory of “kidney storing essence, governing bones and generating marrow” originated in Huangdi Neijing, independent of Western anatomical organ cognition (1). The TCM kidney is a multi-functional unit integrating reproduction, neuroendocrine immunity and skeletal metabolism; insufficient kidney essence directly leads to trabecular microstructural damage and bone loss (4). RNA-seq profiling of kidney-yang deficient rats confirms coordinated dysregulation of kidney, bone, gonad and thyroid gene networks, revealing multi-organ synergy behind this doctrine (5). Network pharmacology and metabolomics studies verify Zuogui Pill, Gushudan and kidney-tonifying monomers target PI3K-AKT and Wnt/BMP pathways to rebalance osteoblast and osteoclast activity (6, 22). Gushudan reshapes intestinal flora and short-chain fatty acid metabolism to protect bone via the gut-kidney axis (23). Cornus officinalis and epimedium-derived ingredients improve osteogenic differentiation (5, 19). Nevertheless, most related research remains at preclinical levels without standardized clinical transformation specifications (19).

### 2.2 Ancient greek humoral theory and renal-skeletal correlation

Hippocrates and Galen’s humoral balance doctrine connects renal excretion function with bone growth and repair. They believed abnormal waste metabolite retention caused by impaired renal filtration disrupts bone mineral deposition, forming the earliest Western empirical association between excretory viscera and bone tissue (2). However, this system only describes body fluid balance, without systematic elaboration of hormone and marrow feedback mechanisms (2).

### 2.3 Ayurvedic Asthi Dhatu theory: excretory viscera and bone homeostasis

Ayurvedic medicine divides human tissues into seven dhatus, among which Asthi Dhatu (bone tissue) relies on balanced visceral metabolite clearance to maintain matrix synthesis; impaired waste excretion results in bone fragility (2). Its intervention system centers on herbal diet conditioning, without complete mineral hormone regulatory frameworks (2).

### 2.4 Comparative analysis of homologies and cognitive divergences

#### 2.4.1 Shared holistic homology

All three ancient medical civilizations independently put forward that visceral function dominates skeletal homeostasis, rejecting isolated local bone pathogenesis, and formed consistent therapeutic logic of regulating viscera to treat bone lesions (2). These empirical records lay the primitive theoretical prototype of modern multi-organ endocrine crosstalk.

#### 2.4.2 Core cognitive divergences

TCM constructs a complete neuroendocrine multi-axis regulatory network with abundant compound herbal multi-target intervention experience (1, 6).

Ancient Greek humoral theory merely describes fluid metabolic disorders, without involving marrow and endocrine feedback regulation;

Ayurveda focuses on dietary conditioning, lacking systematic cognition of calcium-phosphate mineral metabolism.

At present, unified interpretive frameworks mapping TCM “kidney essence” connotation to FGF23/VDR molecular signaling are still absent (2).

Chapter 3 Remodeling Kidney-Bone Cognition via Modern Physiology and Pathology.

### 3.1 Early clinical identification of uremic osteodystrophy

Clinicians systematically summarized unique skeletal complications of end-stage renal failure from the late 19th century, including intractable bone pain and spontaneous pathological fractures, collectively named renal osteodystrophy (ROD) (7). Cohort data confirmed progressive glomerular damage independently triggers bone turnover disorders irrelevant to primary orthopedic lesions (9). Iliac crest bone histomorphometry is recognized as the diagnostic gold standard, subdividing ROD into four subtypes with distinct incidence among dialysis and non-dialysis populations (24). Long-term uncontrolled severe SHPT may progress to multifocal uremic tumoral calcinosis with irreversible joint dysfunction (2). Refractory pruritus in hemodialysis patients is positively correlated with intact PTH and bone turnover markers, reflecting systemic mineral disturbance injury (25).

### 3.2 Vitamin D metabolism: renal endocrine paradigm transformation

Renal proximal tubular CYP27B1 acts as the rate-limiting enzyme converting inactive hepatic 25(OH)D into bioactive calcitriol (8). Renal injury inhibits CYP27B1 transcription, reducing calcitriol synthesis and inducing hypocalcemia, phosphate retention and parathyroid hyperplasia (8). VDR is widely expressed in osteoblasts, renal tubules and parathyroid tissue to mediate multi-organ negative feedback regulation (26). Parathyroid tissue differential CaSR and VDR expression patterns determine individual sensitivity to calcium and vitamin signals, forming the core histopathological basis of primary and secondary hyperparathyroidism (27). Calcitriol upregulates RANKL and downregulates OPG in osteoblasts to balance bone resorption and formation, while directly inhibiting PTH gene transcription to delay gland hyperplasia (28, 29). Renal damage eliminates this inhibitory signal and accelerates diffuse-to-nodular parathyroid proliferation (8). Gene knockout and anephric patient studies revise the exclusive renal activation theory: intestinal and splenic tissues express low levels of functional CYP27B1 to partially compensate calcitriol production, yet the compensation cannot reverse systemic mineral disorder (30, 31). Vitamin D efficacy varies across CKD stages: oral nutritional vitamin only benefits stage1–3 patients, while dialysis populations require VDR activator intervention (16). The vitamin D metabolite ratio (VMR) outperforms single 25(OH)D detection, but unified stage-specific clinical thresholds have not been formulated (16). In diabetic nephropathy, concurrent vitamin D deficiency induces renal tubular ferroptosis via JUN/ATF3 pathways and aggravates bone loss (32).

### 3.3 Secondary hyperparathyroidism: core renal-bone pathological link

PTH transmits renal mineral disturbance to bone tissue mainly through the canonical OPG/RANKL/RANK cascade (28). Intermittent physiological low-dose PTH exerts trabecular anabolic effects; sustained high PTH in SHPT continuously elevates the RANKL/OPG ratio to accelerate cortical bone resorption (28). Iliac intratrabecular tunneling remodeling is a unique histopathological marker of long-term excessive PTH stimulation (28). Accumulated uremic toxins and downregulated skeletal VTH/PTH1R cause skeletal PTH resistance, producing two opposite bone pathological phenotypes: high-turnover osteitis fibrosa and adynamic bone disease (33). Preoperative intact PTH serves as an integrated biomarker reflecting parathyroid hyperplasia severity, bone turnover activity and drug refractoriness (34). mTORC1 hyperactivation under uremic environment drives autonomous parathyroid proliferation; mTOR inhibitors effectively relieve gland overgrowth in animal and renal transplant cohorts (35). Leptin downregulates parathyroid CaSR expression to modulate PTH secretion, forming an obesity-associated auxiliary regulatory axis (35, 36).

### 3.4 Uremic toxins as independent bone injury mediators

Prior to the proposal of FGF23-Klotho axis, gut-derived protein-bound retention toxins were identified as bone damage factors independent of calcium, phosphorus and PTH imbalance (7). Indoxyl sulfate and p-cresyl sulfate enter bone tissue via circulation and activate the AhR pathway to suppress osteogenic gene expression and upregulate Wnt antagonists Sost/Dkk1 (7). Single-toxin injury can be partially reversed by exogenous PTH, while mixed multiple toxins cause irreversible skeletal damage (37). Uremic toxins destroy osteocyte mitochondrial structure and block mitophagy to accumulate reactive oxygen species and inhibit mineralization capacity (25). Intestinal adsorbent AST-120 and mitochondrial-targeted antioxidants restore mitophagy and alleviate bone loss in preclinical models (25). Lanthionine and other retained toxins activate BMP2/RUNX2 cascades to trigger vascular and skeletal ectopic mineralization, forming a toxin-bone-vascular composite injury loop (9). This toxin-centered pathological pathway complements the classic mineral-PTH cascade and lays a theoretical foundation for gut-kidney-bone multi-organ research (23).

## 4 Chapter 4 modern molecular endocrine axes of kidney-bone interaction

### 4.1 Canonical FGF23–Klotho core signaling axis

The discovery of FGF23-Klotho ternary complex built the first bidirectional bone-to-kidney endocrine feedback loop at molecular resolution (10). Osteocyte-secreted FGF23 inhibits renal NaPi phosphate transporters and CYP27B1 transcription; membrane-bound α-Klotho is an indispensable co-receptor for FGFR signal transduction (11, 12). Under physiological steady state, calcitriol and FGF23 maintain negative feedback balance; CKD-induced Klotho depletion breaks homeostasis and triggers massive compensatory FGF23 over-secretion (3, 10). Excess FGF23 acts on cardiac and vascular tissue through Klotho-independent FGFR4 signaling to induce myocardial hypertrophy and medial vascular calcification (3). Moderate aerobic and HIIT training upregulates renal Klotho expression and reduces circulating FGF23 to relieve bone and cardiac lesions in animal models (3). PAI-1 antagonists alleviate FGF23-mediated hypophosphatemia by inhibiting lysosomal degradation of phosphate transporters (38, 39). Most mechanistic data derive from rodent experiments; large prospective human cohorts are required to verify whether FGF23/Klotho targeted intervention reduces fracture and cardiovascular mortality risks (10).

### 4.2 Bidirectional vitamin D endocrine feedback loop

Hepatic cholecalciferol is hydroxylated into inactive 25(OH)D, which relies on renal CYP27B1 to generate bioactive calcitriol, while renal CYP24A1 mediates calcitriol catabolism to maintain metabolic homeostasis (8, 40). VDR distributes widely in osteoblasts, renal tubules and parathyroid cells to realize multi-organ feedback regulation (26). Calcitriol upregulates RANKL and downregulates OPG in osteoblasts to balance bone turnover, and directly inhibits PTH gene transcription to delay parathyroid hyperplasia (29). Renal injury removes this inhibitory signal and accelerates nodular gland proliferation (8). Intestinal and splenic CYP27B1 can partially compensate calcitriol synthesis in anephric patients, yet the compensatory capacity cannot reverse systemic mineral disorder (30). IGF1 upregulates renal CYP27B1 expression to synergistically improve bone mineralization (41). Genomic enhancer studies clarify that PTH-SIK signaling axis controls transcriptional expression of CYP27B1/CYP24A1 to regulate vitamin D activation efficiency (29, 42). The vitamin D metabolite ratio (VMR) has superior diagnostic value over single 25(OH)D detection, but unified CKD stage-specific clinical cutoffs have not been established (16).

### 4.3 OPG/RANKL/RANK and PTH-mediated bone remodeling signaling

PTH regulates bone remodeling mainly through the OPG/RANKL/RANK canonical cascade (28). Intermittent low-dose physiological PTH promotes osteoblast proliferation and trabecular bone formation; sustained high PTH concentration in SHPT increases RANKL secretion to accelerate osteoclast precursor differentiation and cortical bone resorption (28). Intratrabecular tunneling remodeling is a unique histopathological marker of long-term excessive PTH stimulation (28). Uremic toxin accumulation and reduced skeletal VDR/PTH1R expression cause skeletal PTH resistance, forming two opposite bone turnover phenotypes (33). Preoperative intact PTH comprehensively reflects parathyroid hyperplasia, bone turnover level and drug treatment refractoriness (34). mTORC1 overactivation under uremic conditions drives autonomous parathyroid proliferation; mTOR inhibitors relieve gland overgrowth in animal and transplant patient cohorts (35). Leptin downregulates parathyroid CaSR expression to participate in auxiliary PTH secretion regulation, forming an obesity-related regulatory axis (35).

### 4.4 Novel multi-organ crosstalk axes expanding kidney-bone network

#### 4.4.1 Gut microbiota–kidney–bone tripartite axis

Intestinal dysbiosis reduces short-chain fatty acid production and elevates intestinal permeability, leading to massive translocation of indoxyl sulfate, p-cresyl sulfate and TMAO into systemic circulation (13, 43). These metabolites bind osteocytic AhR to inhibit osteogenic gene transcription and upregulate Wnt suppressors Sost/Dkk1, simultaneously restraining bone formation and accelerating resorption (7). Single-toxin damage can be partially reversed by PTH, while mixed multiple toxins induce irreversible skeletal injury (37). Fecal microbiota transplantation, probiotic supplementation and oral adsorbent AST-120 reduce serum toxin concentrations and improve bone mineral density in CKD animal models (13). However, standardized probiotic strains, dosage and intervention cycles for human clinical trials have not been formulated (44). Metabolomics research confirms p-cresyl sulfate disrupts osteoblast glutathione metabolism to inhibit mineralization capacity (37).

#### 4.4.2 Exosome-mediated inter-tissue communication

Extracellular exosomes secreted by renal tubular epithelial cells and osteocytes carry microRNAs and proteins to realize long-distance organ signal exchange (15). Uremic renal exosomes deliver oxidative stress mediators to inhibit mesenchymal stem cell osteogenic differentiation; bone-derived exosomes rich in FGF23 act on renal tubules to suppress phosphate reabsorption and CYP27B1 expression (15). Exosomal signal transmission amplifies the FGF23-Klotho vicious cycle; all relevant research remains at cell and animal model stages, lacking large clinical cohort verification of exosome-derived biomarkers (15, 45).

#### 4.4.3 O-GlcNAc modification and shared heart-kidney-bone pathogenesis

O-GlcNAc protein post-translational modification integrates glucose metabolism and mineral hormone signal transduction (14). Uremia-induced hyperglycemia and metabolic disturbance elevate global O-GlcNAc levels in renal and bone cells, downregulating VDR and FGFR-Klotho signaling to aggravate calcium-phosphate imbalance (14). Abnormal O-GlcNAc modification simultaneously promotes vascular smooth muscle osteogenic transformation, cardiac fibrosis and osteoporosis, explaining synchronous high risk of cardiovascular events and fractures in CKD patients (14). Moderate aerobic exercise reverses excessive O-GlcNAc modification to restore balanced FGF23 and vitamin D signaling, providing a safe non-pharmacological intervention target for combined skeletal and cardiovascular complications (3).

## 5 Chapter 5 clinical translation, intervention strategies and research barriers

### 5.1 Western standardized interventions for CKD-MBD & clinical controversies

#### 5.1.1 Mineral-regulating & vitamin-related drugs

Phosphate binders are first-line basic therapy for hyperphosphatemia (43). Calcium-containing binders effectively reduce serum phosphorus but increase long-term vascular calcification risk; ferric citrate improves renal anemia with prominent gastrointestinal adverse reactions (43). Oral nutritional cholecalciferol only exerts limited efficacy in mild CKD patients; once renal enzymatic activity declines significantly, calcitriol and selective VDR activators become mandatory to inhibit excessive PTH secretion (8). However, VDRA treatment inevitably elevates circulating FGF23 concentration, forming a therapeutic contradiction between parathyroid control and cardiovascular-skeletal toxicity (10). Calcimimetics enhance parathyroid CaSR sensitivity to reduce PTH secretion without stimulating FGF23 overproduction, suitable for combined regimens with VDRAs (46, 47).

#### 5.1.2 Bone-targeted & novel renoprotective agents

Denosumab and zoledronic acid are mainstream anti-resorptive drugs with distinct safety risks across renal function strata (18). Denosumab significantly increases severe hypocalcemia risk in stage4–5 CKD patients, while bisphosphonates accumulate in uremic bone tissue to induce adynamic bone disease (18). SGLT2 inhibitors exert dual skeletal effects: mild CKD cohorts show improved bone mineral density via anti-inflammatory and anti-oxidative pathways, while long-term follow-up of advanced renal failure patients observes elevated fracture incidence with inconsistent subgroup conclusions (17).

#### 5.1.3 Surgical treatment of refractory tertiary hyperparathyroidism

Parathyroidectomy is radical therapy for tumoral calcinosis, intractable bone pain and drug-resistant tertiary hyperparathyroidism (34). Postoperative hungry bone syndrome characterized by transient severe hypocalcemia frequently occurs; long-term follow-up finds fluctuating FGF23/Klotho levels and recurrent SHPT in partial patients (24). Preoperative intact PTH can predict surgical efficacy and postoperative bone metabolic fluctuation to guide optimal operation timing (34).

### 5.2 Translational research of kidney-tonifying traditional chinese medicine

#### 5.2.1 Classic compound prescriptions & multi-omics mechanisms

Zuogui Pill, Gushudan and Erzhi Pill are the most widely validated kidney-tonifying formulas for CKD-MBD intervention (20). Multi-dimensional metabolomics and 16S rRNA sequencing verify Gushudan reshapes intestinal flora, elevates short-chain fatty acid generation, reduces circulating indoxyl sulfate and upregulates renal Klotho to inhibit compensatory FGF23 over-secretion (23). Network pharmacology combined with cell experiments confirms Zuogui Pill activates the PI3K-AKT pathway to alleviate iron overload-induced osteoblast apoptosis (22). Modified Yishen Decoction regulates FGF23/P38MAPK/Wnt cascades to relieve renal interstitial fibrosis and balance bone turnover (32). Combined Chinese-Western therapy shows superior bone-protective effects compared with monotherapy in animal trials, yet large multi-center long-term RCT evidence is insufficient (20).

### 5.2 Bioactive herbal monomers

Icariin, morroniside and fucoidan exert multi-pathway regulatory effects on the kidney-bone axis (32). Fucoidan restores renal Klotho expression and suppresses FGF23 transcription to relieve CKD skeletal lesions (48); psoralea polysaccharides enhance VDR signaling to promote mesenchymal stem cell osteogenic differentiation (49). At present, most monomer research stays at small preclinical experiments, lacking unified clinical dosage, treatment cycle and long-term safety evaluation standards (19).

### 5.3 Preclinical emerging intervention strategies

#### 5.3.1 Gut microbiota targeted therapy

Probiotics, oral adsorbent AST-120 and fecal microbiota transplantation reduce circulating protein-bound uremic toxins, relieve AhR-mediated osteogenic suppression and normalize PTH/FGF23 levels in CKD animal models (13). However, human cohort verification data are insufficient, without unified strain matching specifications (44).

#### 5.3.2 Non-pharmacological exercise intervention

Moderate aerobic and HIIT training upregulates renal Klotho and reduces circulating FGF23, balancing serum phosphate, PTH and active vitamin D simultaneously (3). Resistance training elevates IGF-1 myokine secretion to synergistically promote bone formation, a low-cost adjuvant therapy without mineral disturbance risks (11). Stratified exercise intensity and cycle protocols for dialysis and non-dialysis populations still require prospective clinical standardization.

#### 5.3.3 Novel molecular targeted drugs

Anti-FGF23 neutralizing antibodies and recombinant soluble Klotho enter preclinical and early clinical development (10). Anti-FGF23 agents correct renal phosphate wasting but carry hyperphosphatemia risks in specific subgroups; recombinant Klotho exhibits bone and cardiovascular protective effects, yet its half-life and tissue targeted delivery system need further optimization (50).

### 5.4 Core clinical translation bottlenecks

#### 5.4.1 Deficiencies of bone biomarkers

Conventional serum calcium, phosphorus and bone alkaline phosphatase cannot distinguish high-turnover and adynamic bone disease (25). Novel markers including FGF23, soluble Klotho and VMR have superior predictive performance for fractures and vascular calcification, yet unified detection standards and age/CKD stage-specific reference intervals are absent (16). Iliac crest biopsy remains the gold standard but is limited by invasiveness; ¹^8^F-NaF PET realizes non-invasive bone turnover evaluation with high equipment cost barriers (51).

#### 5.4.2 Stratified diagnosis & treatment gaps

Current KDIGO guidelines lack differentiated management plans for children, elderly, diabetic nephropathy and renal transplant patients (25). Most clinical trials exclude moderate-severe renal insufficiency patients, leading to insufficient dosage adjustment evidence for stage3–5 subgroups (19). Sarcopenia, frailty and diabetes synergistically aggravate bone fragility, while composite intervention protocols have not been systematically formulated (33).

#### 5.4.3 Disconnection between basic & clinical research

Most conclusions of exosome, O-GlcNAc and uremic toxin research only rely on cell/rodent data without human cohort validation (14). Multi-omics differential markers screened in animal models cannot be stably replicated in large patient populations (13). Kidney-tonifying herbal research mainly consists of small single-center trials lacking long-term fracture/mortality follow-up endpoints (52).

## 6 Chapter 6 summary, controversies & future integrated research prospects

### 6.1 Full-text general summary

Horizontally, TCM kidney-bone theory, ancient Greek humoral doctrine and Ayurvedic Asthi Dhatu all adopt holistic visceral-skeletal thinking, yet form differentiated interpretation and intervention systems, providing embryonic prototypes for modern multi-organ endocrine crosstalk (1, 2). Vertically, modern biomedical research evolves through three successive phases:

Pathological observation stage: Identify ROD and establish phosphate-vitamin D-SHPT pathological cascade (7, 9).

Core molecular endocrine stage: Discover FGF23-Klotho bidirectional feedback and FGF23/vitamin D/PTH interactive network (10, 11).

Multi-organ expansion stage: Clarify gut, exosome and O-GlcNAc mediated inter-tissue communication pathways (33, 44).

Clinically, two parallel intervention systems have formed: Western single-target precise therapy and TCM multi-component holistic regulation (7, 20). This review systematically sorts all diagnostic and translational bottlenecks of CKD-MBD.

### 6.2 Unresolved core research controversies

#### 6.2.1 Molecular mechanism disputes

Dual identity of FGF23: Physiological FGF23 maintains mineral balance, while pathological excess FGF23 independently induces cardiac hypertrophy and vascular calcification; academia debates whether elevated FGF23 is merely compensatory feedback or an independent pathogenic driver, with contradictory phosphate disturbance risks of anti-FGF23 therapy across subgroups (3, 10);

VMR clinical threshold dispute: Low 25(OH)D correlates with high fracture risk in mild CKD, while the correlation disappears after confounding factor adjustment; unified global clinical cutoffs have not been reached (16).

The precise molecular switch determining divergent PTH anabolic/catabolic effects under uremic toxin accumulation and skeletal PTH resistance remains unclear (28).

### 6.2 Clinical therapeutic controversies

SGLT2 inhibitors and denosumab show inconsistent skeletal safety across renal function strata, without unified combined medication schemes (17, 18).

Integrated Chinese-Western therapy lacks large multi-center RCTs with fracture/cardiovascular hard endpoints, without standardized compound matching rules (20).

Preclinical effective microbiota/exercise/recombinant Klotho interventions cannot be transformed into unified human clinical regimens (3, 50).

### 6.3 Cross-cultural research defects

Existing literatures separate TCM kidney essence theory and FGF23/VDR molecular signaling, lacking unified interpretive frameworks linking ancient holism and modern mineral endocrinology (44).

### 6.3 Future integrated research directions

#### 6.3.1 Multi-omics precision biomarker construction

Integrate transcriptomics, metabolomics and microbiomics to build composite biomarker panels combining FGF23, soluble Klotho and VMR; establish age/gender/CKD-stage reference ranges to realize early screening before calcium-phosphate abnormalities (16, 21, 53). Further explore sclerostin and activin A as auxiliary markers to distinguish bone turnover subtypes (28, 54).

#### 6.3.2 Deep exploration of multi-organ crosstalk mechanisms

Clarify complete causal chain of gut flora-tryptophan metabolite-AhR-FGF23/Klotho via large human cohort research (13, 55).

Elaborate integrated signal cascades of O-GlcNAc, mTOR and exosome linking heart, kidney and bone, develop tissue-targeted vesicle delivery tools (33, 56, 57).

Conduct long-term cohort research on sarcopenia and kidney-bone axis aging interaction (21).

#### 6.3.3 Integrated Chinese-Western individualized intervention research

Combine network pharmacology and multi-omics animal validation to map multi-target regulatory networks of kidney-tonifying formulas on FGF23/VDR/RANKL pathways, clarify synergistic advantages of multi-components compared with single Western drugs (7, 20).

Design stratified RCTs grouped by CKD stage and bone turnover subtype, take fracture and cardiovascular events as primary endpoints to fill clinical evidence gaps (20).

Optimize herbal monomer sustained-release preparations combined with low-dose calcitriol to reduce mineral adverse reactions (32).

### 6.4 Novel target & non-pharmacological transformation

Promote clinical translation of anti-FGF23 antibody and recombinant Klotho; formulate stratified exercise intensity standards for dialysis and non-dialysis patients; unify probiotic strain, dosage and treatment cycle specifications (3, 10, 58).

### 6.5 Cross-cultural theoretical fusion research

Construct unified interpretive system integrating three ancient medical holistic theories and modern kidney-bone endocrine network; systematically compare ideological similarities and divergences; excavate potential intervention targets from ancient herbal formulas based on mineral metabolism molecular perspective (1, 44).

### 6.6 Stratified clinical guideline optimization

Carry out subgroup trials targeting children, elderly, diabetic nephropathy and transplant patients, formulate differentiated biomarker monitoring thresholds and hierarchical intervention plans to supplement special population recommendations for revised KDIGO guidelines (25, 51, 59).

## 7 Concluding remarks

Bidirectional kidney-bone endocrine crosstalk acts as a bridge connecting ancient empirical medicine and modern molecular endocrinology. This review systematically sorts its cross-cultural origins and chronological evolution, summarizes core molecular cascades and clinical intervention regimens, and elaborates unresolved controversies and translational bottlenecks. Future research needs to break the isolation between TCM and modern biomedicine, narrow the gap between preclinical animal experiments and human cohort evidence, develop multi-omics precise biomarkers and integrated Chinese-Western individualized therapy, to provide reliable theoretical and clinical references for the prevention and treatment of CKD-MBD and related metabolic bone diseases.

Unified Reference List (Continuous 1–59, All Matched with In-Text Citations, Frontiers Standard Format).

## Data Availability

The original contributions presented in the study are included in the article/supplementary material. Further inquiries can be directed to the corresponding author.

## References

[1] Ju D Liu M Zhao H Wang J . Mechanisms of "kidney governing bones" theory in traditional Chinese medicine. Front Med. (2014) 8:389–93. doi: 10.1007/s11684-014-0362-y 25204291

[2] Harris MJ Pedraza Cruz P Junejo S . Multifocal uremic tumoral calcinosis from prolonged hyperparathyroidism in ADPKD. Cureus. (2026) 18:e108102. doi: 10.7759/cureus.108102 42226858 PMC13222623

[3] Rokhsati S Shahsavari N Rabbani S Suzuki K Khoramipour K . High-Intensity Interval and Aerobic Training Alleviate Cardiac Pathology, Apoptosis, and Atrial Fibrillation in Rats with Chronic Kidney Disease: The Roles of FGF23 and Klotho. Biomolecules. (2026) 16:513. doi: 10.3390/biom16040513 42072635 PMC13113790

[4] Zhu H Liu Q Li W Huang S Zhang B Wang Y . Biological Deciphering of the "Kidney Governing Bones" Theory in Traditional Chinese Medicine. Evid Based Complement Alternat Med. (2022) 2022:1685052. doi: 10.1155/2022/1685052 35392645 PMC8983196

[5] Yang S Wang T Zhang J Leng X Yao B . Integrated RNA-Seq Analysis Uncovers the Potential Mechanism of the "Kidney Governing Bones" Theory of TCM. Evid Based Complement Alternat Med. (2022) 2022:7044775. doi: 10.1155/2022/7044775 35399624 PMC8986393

[6] Tong X Yang Y Gong S Han X Wu Y Ma X . Data Mining and Network Pharmacology Analysis of Kidney-Tonifying Herbs on the Treatment of Renal Osteodystrophy Based on the Theory of "Kidney Governing Bones" in Traditional Chinese Medicine. Evid Based Complement Alternat Med. (2022) 2022:1116923. doi: 10.1155/2022/1116923 36238608 PMC9552684

[7] Russell CR 3rd Chen N Biruete A Allen M Metzger C Wallace JM . Gut-derived uremic toxin indoxyl sulfate and aryl hydrocarbon receptor in renal osteodystrophy. Biochimie. (2026) 248:40–7. doi: 10.1016/j.biochi.2026.05.009 42214784

[8] Díaz-Tocados JM Rodríguez-Ortiz ME Herencia C López-Baltanás R Jurado-Montoya D García-Saez RM . Comparative effects of calcitriol and calcimimetic on bone health in renal insufficiency. FASEB J. (2024) 38. doi: 10.1096/fj.202302704R 38847773

[9] Christodoulou M Aspray TJ Piec I Fraser WD Schoenmakers I VDOP Trial group . Alterations in regulators of the renal-bone axis, inflammation and iron status in older people with early renal impairment and the effect of vitamin D supplementation. Age Ageing. (2024) 53:afae096. doi: 10.1093/ageing/afae096 38770543 PMC11106582

[10] Waitupu A Pratiwi L Sutanto H Santoso D Hertanto DM . Molecular pathophysiology of chronic kidney disease-mineral and bone disorder: Focus on the fibroblast growth factor 23-Klotho axis and bone turnover dynamics. Exp Physiol. (2025). doi: 10.1113/EP092401 40014035 PMC13394114

[11] Guldan M Ozbek L Fidan DG Narin AE Hansen D Diaz-Tocados JM . Circulating α-Klotho and Multidimensional Aging and Frailty Outcomes: A Systematic Review and Meta-Analysis from the European Renal Association CKD-MBD Working Group. Calcif Tissue Int. (2026) 117:72. doi: 10.1007/s00223-026-01537-3 42060134 PMC13132909

[12] Wilson R Mukherjee-Roy N Gattineni J . The role of fibroblast growth factor 23 in phosphate balance. Pediatr Nephrol. (2024) 39:3439–51. doi: 10.1007/s00467-024-06395-5 38874635

[13] Ouyang Z Hu W Zhu F Zhang Y Wang L Ye Q . The role of gut dysbiosis in endocrine and metabolic derangements of chronic kidney disease: mechanisms, controversies, and future perspectives. Front Endocrinol (Lausanne). (2026) 17:1854704. doi: 10.3389/fendo.2026.1854704 42290869 PMC13261356

[14] Wang S Guo M Yang B Fan T Zhang X Feng C . O-GlcNAcylation: A bridge for regulating the function of the "heart-kidney-bone axis. Biochem Biophys Rep. (2026) 46:102635. doi: 10.1016/j.bbrep.2026.102635 42199508 PMC13199816

[15] Hu X Yang B Chen H Min M Liu N Xue C . Heterogeneous regulation of fibroblast growth factor 23 in acute kidney injury, chronic kidney disease, and polycystic kidney disease: mechanisms, diagnostic utility, and clinical implications. Front Mol Biosci. (2026) 13:1764206. doi: 10.3389/fmolb.2026.1764206 41783140 PMC12953371

[16] Ji Y Bi R Wang L . Research on vitamin D metabolic regulation in the pathogenesis of related diseases. Front Endocrinol (Lausanne). (2026) 17:1762302. doi: 10.3389/fendo.2026.1762302 41928887 PMC13038531

[17] Cassani de Oliveira M Rufino L Mendes Lourenço AL Corrêa EA Mac-Way F Pelepenko LE . Effects of gliflozins on bone health of patients with chronic kidney disease: a systematic review. J Nephrol. (2026). doi: 10.1093/joneph/aajag035 42301927

[18] Xiao R Faucon AL He N Javaid MK Ji D Xu Y . Risks of Hypocalcemia and Other Bone Mineral Disorders for Denosumab Versus Zoledronate Across the Spectrum of Kidney Function: A Target Trial Emulation. Am J Kidney Dis. (2026) 88:88–98. doi: 10.1053/j.ajkd.2026.02.642 42009245

[19] Zheng Z Yang F Ye X Jin Y Zhu H Zhou X . Molecular mechanisms of polysaccharide components of kidney-tonifying traditional Chinese medicine in regulating osteoporosis. Front Pharmacol. (2026) 17:1685724. doi: 10.3389/fphar.2026.1685724 42272846 PMC13248022

[20] Ren M Wang Y Yuan Y Du H Liang Q Qin F . Integration of UHPLC-MS and mass spectrometry imaging techniques revealed the protective mechanism of Gushudan in postmenopausal osteoporosis rats via branched-chain amino acid metabolism based on the 'kidney-bone' axis. J Chromatogr B. (2025) 1256:124540. doi: 10.1016/j.jchromb.2025.124540 40023006

[21] Wu Q Wan Z Lan J Zhang X Huang L Ji B . High-throughput UHPLC-MS/MS method for 15 protein-bound uremic toxins in human serum. Clin Chim Acta. (2026) 589:121026. doi: 10.1016/j.cca.2026.121026 42044764

[22] Lin Y He Q Chen B Li Z Chen C Deng W . Zuogui Pills alleviate iron overload-induced osteoporosis by attenuating ROS-mediated osteoblast apoptosis via the PI3K-AKT pathway and mitigating mitochondrial damage. J Ethnopharmacol. (2025) 344:119455. doi: 10.1016/j.jep.2025.119455 39971012

[23] Tong L Feng Q Lu Q Zhang J Xiong Z . Combined 1H NMR fecal metabolomics and 16S rRNA gene sequencing to reveal the protective effects of Gushudan on kidney-yang-deficiency-syndrome rats via gut-kidney axis. J Pharm BioMed Anal. (2024) 217:115919. doi: 10.1016/j.jpba.2022.114843 35623116

[24] Lafage-Proust MH Jørgensen HS Bravenboer N Ferreira A Bégin MJ Cannata-Andia J . Bone histomorphometry for the diagnosis of renal osteodystrophy - a European consensus statement. Bone. (2025) 199:117544. doi: 10.1016/j.bone.2025.117544 40446949

[25] Tschirner A Weber H Schermilly K Böckmann I Pekic M Kanzelmeyer N . Markers of Mineral Metabolism in Children With CKD Stages 2 to 5D. Kidney Int Rep. (2026) 11:103800. doi: 10.1016/j.ekir.2026.103800 41767622 PMC12938118

[26] Kamboj K Kumar V Yadav AK . *In Vitro* Study of Vitamin D Effects on Immune, Endothelial, and Vascular Smooth Muscle Cells in Chronic Kidney Disease. Int J Mol Sci. (2025) 26:3967. doi: 10.3390/ijms26093967 40362207 PMC12071356

[27] Blanchard B Hadjadj S Tang E Kervadec J Ouchelouche S Frere P . Parathyroid histology in normocalcemic and hypercalcemic primary hyperparathyroidism with hypercalciuric renal stones. J Endocr Soc. (2026) 10:bvag014. doi: 10.1210/jendso/bvag014 41907687 PMC13026408

[28] Thomsen LK Laursen KS Sikjær T Thomsen JS Andreasen CM Loundagin L . Intratrabecular tunneling remodeling – a previously overlooked mode of remodeling hyperactivated by PTH. J Bone Miner Res. (2026). doi: 10.1093/jbmr/zjag081 42119027

[29] Yoon SH Meyer MB Arevalo C Tekguc M Zhang C Wang JS . Title incomplete in source. J Clin Invest. (2023) 133:e163627. doi: 10.1172/jci163627 36862513 PMC10145948

[30] Lee SM Cichanski SR Pintozzi NG Kaufmann M Jones G Meyer MB . Kidney deletions of Cyp27b1 fail to reduce serum 1,25(OH)2D3. J Steroid Biochem Mol Biol. (2025) 250:106734. 10.1016/j.jsbmb.2025.106734 PMC12496027 40096920

[31] Harada S Abe M Ono Y Ishida K Tojo A . Renal Sarcoidosis With Increased 1,25(OH)2D3 Production via CYP27B1-Expressing Epithelioid Granuloma: A Case Report and Review. Cureus. (2025) 17:e95419. doi: 10.7759/cureus.95419 41306174 PMC12645163

[32] Yu W Zeng C Wang C Jia S Liu C Zeng Y . N1F(Improved-Nephropathy 1 Formula) Ameliorates Renal Interstitial Fibrosis via Inhibiting Extracellular Matrix Deposition and Regulating the FGF23/P38MAPK/Wnt Pathway. Cell Biochem Biophys. (2024) 82:927. doi: 10.1007/s12013-024-01244-6 38722471

[33] Bover J Arana C Ureña P Torres A Martín-Malo A Fayos L . Hyporesponsiveness or resistance to the action of parathyroid hormone in chronic kidney disease. Nefrologia (Engl Ed). (2021) 41:514–28. doi: 10.1016/j.nefroe.2021.11.014 36165134

[34] Liao MT Wu CC Hou YC Tsai KW Shih LJ Lu KC . Pre-parathyroidectomy PTH as an integrated biomarker of glandular remodeling and skeletal turnover in secondary hyperparathyroidism. Int J Mol Sci. (2026) 27:5094. doi: 10.3390/ijms27115094 42278618 PMC13258313

[35] Naveh-Many T Brook E Hassan A . New regulators of parathyroid hormone synthesis and secretion. Curr Opin Nephrol Hypertens. (2026) 35:419–26. doi: 10.1097/MNH.0000000000001190 42199077

[36] Crespo Trevino R Sari E Saldanha Lima C Vercollone J . Macrophage Activation in Lupus Nephritis: An Unknown Cause of Severe Hypercalcemia. R I Med J (2013). (2025) 108:14–6. 41150620

[37] Wu Q Lan J Wan Z Liang Y Nie K Zhou J . Parallel metabolomic and transcriptomic profiling reveals the cytotoxic mechanism of protein-bound uremic toxin p-cresol sulfate via disruption of glutathione and glycerophospholipid metabolism in MC3T3-E1 osteoblasts. BMC Nephrol. (2026) 27:369. doi: 10.1186/s12882-026-05004-x 42045850 PMC13270783

[38] Qian C Ito N Tsuji K Sato S Kikuchi K Yoshii T . A PAI-1 antagonist ameliorates hypophosphatemia in the Hyp vitamin D-resistant rickets model mouse. FEBS Open Bio. (2024) 14:290–9. doi: 10.1002/2211-5463.13745 38050660 PMC10839342

[39] Küng CJ Haykir B Schnitzbauer U Egli-Spichtig D Hernando N Wagner CA . Fibroblast growth factor 23 leads to endolysosomal routing of the renal phosphate cotransporters NaPi-IIa and NaPi-IIc *in vivo*. Am J Physiol Renal Physiol. (2021) 321:F785–798. doi: 10.1152/ajprenal.00250.2021 34719948

[40] Lu S Gao J Zhang H Fan C Liu Z An Q . Patulin Affects the Hydroxylation of Vitamin D by Downregulating 25-hydroxylase (CYP2R1) and 25-hydroxyvitamin D 1-alpha-hydroxylase (CYP27B1) Expression and Interfering Their Substrate Binding Sites. J Biochem Mol Toxicol. (2026) 40:e70877. doi: 10.1002/jbt.70877 42085557

[41] Díaz Tocados JM Pérez Gómez A Domínguez Coral JD Valdivielso JM . The multiple functions of insulin-like growth factor 1 in kidney disease. Nefrologia (Engl Ed). (2025) 45:501337. doi: 10.1016/j.nefro.2025.501337 41005827

[42] Meyer MB Pike JW . Genomic mechanisms controlling renal vitamin D metabolism. J Steroid Biochem Mol Biol. (2023) 232:106331. doi: 10.1016/j.jsbmb.2023.106252 36657729 PMC10006327

[43] Lei Y Xiong G Lei D Wu H Peng X Chen L . Gut microbiota-derived TMAO drives the kidney-bone-vascular axis in chronic kidney disease complications. Ren Fail. (2025) 47:2575434. doi: 10.1080/0886022x.2025.2575434 41232933 PMC12616656

[44] Wang XQ Zou XR Zhang YC . From "Kidneys Govern Bones" to Chronic Kidney Disease, Diabetes Mellitus, and Metabolic Bone Disorder: A Crosstalk between Traditional Chinese Medicine and Modern Science. Evid Based Complement Alternat Med. (2016) 2016:4370263. doi: 10.1155/2016/4370263 27668003 PMC5030442

[45] Liu X Yu W Huang C Wang Z Qian Y Chen X . Exosomes in bone health and disease: cellular crosstalk, systemic signaling, and AI-driven advances in regenerative therapy. Stem Cell Res Ther. (2026) 17:265. doi: 10.1186/s13287-026-05073-7 42216068 PMC13440132

[46] Arampatzis S Ernandez T Teta D Bonani M Georgalis A Flury S . New insights into the management of secondary hyperparathyroidism in non-dialysis dependent chronic kidney disease: extended release calcifediol. J Nephrol. (2026) 39:443–54. doi: 10.1093/joneph/aajaf008 42227696

[47] Derai A Hansen D Jørgensen HS . Secondary hyperparathyroidism in chronic kidney disease: update on pathophysiology and new treatment targets. Curr Opin Endocrinol Diabetes Obes. (2026) 33:137–45. doi: 10.1097/MED.0000000000000961 42083432

[48] Liu BH Chong FL Yuan CC Liu YL Yang HM Wang WW . Corrigendum: Fucoidan ameliorates renal injury-related calcium-phosphorus metabolic disorder and bone abnormality in the CKD-MBD model rats by targeting FGF23-Klotho signaling axis. Front Pharmacol. (2024) 15:1446609. doi: 10.3389/fphar.2024.1446609 39108753 PMC11301053

[49] Liu CL Li MR Long J Qian ST Zhang C Li P . AI-driven discovery of synergistic combinations from Psoralea corylifolia fruit for treating osteoporosis. Phytomedicine. (2026) 159:158427. doi: 10.1016/j.phymed.2026.158427 42308991

[50] Yoon SH Passarella P . Recurrent Soft Tissue Infections Associated With Burosumab Therapy in X-Linked Hypophosphatemic Rickets. JCEM Case Rep. (2023) 1:luad120. doi: 10.1210/jcemcr/luad120 37954837 PMC10634627

[51] Yang Q Chen S Yu Z Wen X Ou S . Advances in noninvasive diagnosis of renal osteodystrophy. Ren Fail. (2025) 47:2593710. doi: 10.1080/0886022X.2025.2593710 41309376 PMC12667297

[52] Yin L Chen X Xu J . Exploring the ROS-Related Therapeutic Mechanism of Bushen Huoxue Decoction in Postmenopausal Osteoporosis through Transcriptomics and Network Pharmacology. OMICS. (2026) 30:436–48. doi: 10.1177/15578100261458418 42275005

[53] Fernández-Villabrille S Martín-Carro B Martín-Vírgala J Rodríguez-Santamaria MDM Baena-Huerta F Muñoz-Castañeda JR . Novel Biomarkers of Bone Metabolism. Nutrients. (2024) 16:605. doi: 10.3390/nu16050605 38474734 PMC10935093

[54] Derwich W Feć K Gawda A Kopa K Kopeć J Nowak I . Osteocalcin Beyond Bone: Molecular Mechanisms, Endocrine Networks, and Translational Perspectives Across Metabolism, Neurobiology, and Chronic Disease. Int J Mol Sci. (2026) 27:2992. doi: 10.3390/ijms27072992 41977179 PMC13074106

[55] Wang Q Zhou Z Pang L Du Y Li X Dai L . Gut microbiota in chronic kidney disease-mineral and bone disorder. Front Endocrinol (Lausanne). (2026) 17:1802845. doi: 10.3389/fendo.2026.1802845 42051461 PMC13110955

[56] Duan Y Zhao LJ Lu YT Li J Li SX . Crosstalk between kidney and bones: New perspective for modulating osteoporosis. Ageing Res Rev. (2025) 109:102776. doi: 10.1016/j.arr.2025.102776 40389172

[57] Wen R Wang H Wang S . Deciphering inter-organ communication: The multi-organ-bone axis in osteoporosis. J Orthop Translat. (2026) 58:101119. doi: 10.1016/j.jot.2026.101119 42100504 PMC13147401

[58] Zhao X Zhai Q Jia X Zhu Y Bao Y Min X . Supplementation with the probiotic Bifidobacterium lactis BB-12 improves the efficacy of alendronate sodium/vitamin D3 therapy on skeletal health in stage 3 CKD. J Endocrinol Invest. (2026) 49:1985–96. doi: 10.1007/s40618-026-02877-5 41989512

[59] Haarhaus M Aaltonen L Cejka D Cozzolino M de Jong RT D'Haese P . Management of fracture risk in CKD-traditional and novel approaches. Clin Kidney J. (2022) 16:456–72. doi: 10.1093/ckj/sfac230 36865010 PMC9972845

